# A holistic approach to predicting diabetes risk via biomarkers

**DOI:** 10.1016/j.ebiom.2021.103563

**Published:** 2021-08-26

**Authors:** Alan A. Cohen

**Affiliations:** a PRIMUS Research Group, Department of Family Medicine, University of Sherbrooke, 3001, 12e avenue Nord, Sherbrooke, Quebec J1H 5N4, Canada; b Research Center on Aging, Sherbrooke, Quebec J1H 4C4, Canada; c Research Center of Centre Hospitalier Universitaire de Sherbrooke, Sherbrooke, Quebec J1H 5N4, Canada

Leo Tolstoy's novel Anna Karenina starts with the famous line, “All happy families are alike; each unhappy family is unhappy in its own way.” This “Anna Karenina Principle” has been applied in numerous contexts, including ecological risk assessment, microbiomes, economics, and many others. In physiology and health, we might rephrase it as a hypothesis; “All healthy physiologies are healthy in relatively standard ways, but there are limitless ways in which physiology can go awry.” This hypothesis raises interesting corollaries: to what extent is there a general, unique profile of physiological “health,” which might be perturbed in a multiplicity of ways? Can we understand health as the body's ability to maintain such a homeostatic state, or more broadly its dynamic equilibrium? Might having an overall profile that is far from average be an indicator either of a specific but undiagnosed pathology, or of a general lack of resiliency predisposing the individual to higher risks of a host of adverse outcomes?

One way to operationalize the Anna Karenina Principle and tackle such questions is via the Mahalanobis distance (MD), a measure that assesses how unusual a multivariate profile is, taking into account known correlations among the variables [1]. In the health context, it can be applied to standard clinical biomarkers [2]. High MD scores (i.e., more unusual profiles) were hypothesized to represent homeostatic dysregulation, and have since been shown to increase with age and predict numerous adverse health outcomes, as well as to be associated with diet and socioeconomic predictors of health [3,4]. This approach has been used in both industrialized and non-industrialized human populations, as well as in various species [5], [6], [7]. Counterintuitively, this approach can generate information that is not available by examining the component biomarkers one at a time – for example, individuals who have clinically normal profiles on every individual biomarker can still have overall profiles that are highly aberrant and suggest high health risk.

In this issue of *EBioMedicine*, Flores-Guerrero et al. [8] break new ground by bringing the MD metric toward the clinical realm. Specifically, using a longitudinal sample of 6,247 non-diabetics in the Netherlands, they asked whether MD scores calculated from 32 circulating biomarkers predicted risk of Type-II diabetes incidence. Across an elegant series of analyses, they consistently found support for this hypothesis. Furthermore, MD appears to be at least partially tapping into information that is not generally included in current diabetes prediction: hazard ratios remained important after adjustment for classical risk factors such as glucose level, obesity, and family history. The non-negligible effect sizes suggest substantial potential to integrate the information into clinical risk algorithms, though much work would remain to identify an optimal biomarker panel, demonstrate superiority to existing algorithms, and validate clinical utility.

Etiologically, some of the sensitivity analyses presented by Flores-Guerrero et al. [8] imply that the MD signal is broadly distributed among many biomarkers rather than specific to a set of cardio-metabolic indicators, making it unlikely that MD is directly detecting metabolic syndrome or related diabetes precursors. Rather, MD may detect a more general physiological dysfunction that can feed into metabolic processes, or an incidental correlate such as a lifestyle-driven dysfunction that could co-vary with diabetes risk. Teasing apart such hypotheses will be important to validate the clinical potential.

Going forward, the work by Flores-Guerrero et al. [8] is likely to represent the tip of the iceberg, both in terms of clinically oriented applications of MD, and for development of multivariate approaches to synthesize relevant underlying physiological processes. More broadly, the Anna Karenina Principle is one expression of a more general shift toward network physiology, network medicine, and complexity science in biomedicine [9]. The underlying model of physiology supposed by the approach is a gentle challenge to the reductionist approaches that tend to dominate biomedical research. Reductionism breaks the biology down into component molecules, cells, and pathways, and looks for highly specific diagnostic or therapeutic molecules. This conventional approach has certainly produced some impressive results, and will continue to do so, but in many domains reductionism has already picked the lowest hanging fruits, and is bumping up against the limits of small effect sizes, contingent results and complex networks of molecules that defy simple characterization or manipulation. For example, Alzheimer's research targeting amyloid-beta has been strikingly unsuccessful, perhaps due to a failure to understand the complexity of amyloid-beta's integration into immune networks and the delicate balance between adaptation and pathology [10]. The broader question posed by Flores-Guerrero et al. [8] is, what might we gain by using integrative rather than compartmentalized ways to study physiology? In both clinical and research contexts, methods such as MD present a way to reconceptualize health in a more integrative fashion and to uncover novel processes that function at the intersection of multiple pathways and systems. Long-term, this will fuel more precise and personalized interventions that account for the ensemble of an individual's dynamic physiology.

## Contributors

AAC is the sole author.

## Declaration of Competing Interest

AAC is founder and CEO at Oken Health.
